# Non-participation in initial and repeated health risk appraisals – a drop-out analysis based on a health project

**DOI:** 10.1186/s12913-019-3949-9

**Published:** 2019-02-21

**Authors:** Wilhelmus Johannes Andreas Grooten, Amanda Hansson, Mikael Forsman, Katarina Kjellberg, Allan Toomingas, Mira Müller, Magnus Svartengren, Björn Olov Äng

**Affiliations:** 1 Department of Neurobiology, Care Sciences and Society, Division of Physiotherapy, Karolinska Institutet, Huddinge, Sweden; 2 Allied Health Professionals Function, Functional area Occupational Therapy and Physiotherapy, Karolinska University Hospital, Stockholm, Sweden; 3 Institute of Environmental Medicine, Karolinska Institutet, Stockholm, Sweden; 4 Centre for Occupational and Environmental Medicine, Stockholm County Council, Stockholm, Sweden; 5 Division of Ergonomics, KTH Royal Institute of Technology, Huddinge, Sweden; 6 Department of Medical Sciences, Uppsala University, Uppsala, Sweden; 7 School of Education, Health and Social Studies, Dalarna University, Falun, Sweden; 8 Center for Clinical Research Dalarna - Uppsala University, Falun, Sweden

**Keywords:** Health promotion, Lifestyle changes, Occupational health services, Physical activity, Participation, Sickness prevention

## Abstract

**Background:**

Health risk assessment (HRAs) are commonly used by occupational health services (OHS) to aid workplaces in keeping their employees healthy, but for unknown reasons, many employees choose not to participate in the HRAs. The aim of the study was to explore whether demographic, lifestyle and health-related factors in employees are associated with non-participation in initial and repeated HRAs.

**Methods:**

In an OHS-based health project, 2022 municipal employees were asked to participate in three repeated HRAs. Multiple logistic regression analyses were used so as to determine associations between non-participating and demographic, lifestyle and health-related factors (e.g. biomarkers).

**Results:**

Among the employees who were asked to participate in the health project, more than half did not participate in any HRA and among those who did, more than one third did not participate in repeated HRAs. Young age, male sex and being employed in the Technical department or Health and Social Care department in comparison with being employed in the department for Childcare and Education were factors significantly associated with non-participation in the initial HRA. These factors, together with being on sick leave and having unhealthy dietary habits, were factors associated with non-participation in repeated HRAs.

**Conclusions:**

Among the non-participators in initial HRAs and in repeated HRAs younger men and those already related to ill-health were overrepresented. This implicates that health care providers to a higher extent should focus on “those most needed” and that employers should be more engaged in results of repeated HRA’s. Future studies should focus on modifiable variables that could make the HRAs more attractive and inclusive.

## Background

Health promotion is the process of enabling people to increase control over and improve their health [1], while disease prevention covers measures not only to prevent the occurrence of disease, such as risk factor reduction, but also to arrest its progress and reduce its consequences once it is established [2]. The workplace is an important arena for both health promotion and disease prevention, since it is possible to reach a vast majority of the adult population. Guidelines for improving the physical fitness of employees are established and the use of health risk appraisals (HRAs) by Occupational Health Services (OHS) is suggested as being an important part of health promotion campaigns for employees [3]. The general goals for OHS are to promote a healthy workplace environment, keeping the employees safe and healthy, and improving their well-being as well as their work ability [4, 5]. For these reasons, most OHS in Sweden use HRAs in order to monitor early signs of illness, poor lifestyle factors (such as a lack of physical activity, high alcohol and tobacco consumption and unhealthy dietary habits), sleep disorders, and increased stress [6–8], by means of self-administered questionnaires. In addition to the questionnaires, several physical measures such as BMI and waist size are often taken along with laboratory tests such as cholesterol level, blood glucose, cardio pulmonary fitness, etc. The goal of using HRAs is to use these measures to provide advice to employees on their current level of health and what they could do to improve it. One common HRA method used by the OHS is the “Health Profile Assessment” (HPA) which is used both as a tool for screening and intervention, and aims to bring about changes in lifestyle habits, perceived health and physiological measurements [6, 9].

Repeated HPAs can be used to evaluate the effectiveness of the HRA and the associated actions. Only a few previous studies have been found that have used repeated HPAs and they showed improvements in lifestyle and health measures [6, 8, 9]. We have learned that there is no explicit strategy for the use of repeated HRA in Sweden. In our reports, interview data with employers indicate rather that employers HRA’s are using HRA for gathering information for work environmental issues, as an appreciation for employees, and as an investment in personal resources, to increase productivity [10]. There seems to be a greater interest in having offered HRA than taking the results further.

One important factor for the effectiveness of HRA is of course participation. The OHS personal needs to motivate the employees to participate in the initial HRA, to perform actions and to participate in repeated HRAs. Literature reviews have shown that usually around one out of three employees participate in HRAs [11, 12]. If participation rates differ between different groups (e.g. only those who are highly motivated participate), the interpretation of the research findings could be difficult due to selection bias [13]. Moreover, increased understanding of factors related to differential rates of participation could lead to the development of more effective health promotion strategies and programmes for the workplace [13]. Less is known about the participation in repeated HRAs. Based on our clinical expectations, it is believed that those employees who participate once are likely more motivated to participate again, especially those with good health and a healthy life-style.

Factors associated with non-participation are lack of time, not receiving the invitation, being a smoker and having poor self-reported health [14], moral issues [15], and high socioeconomic status [13]. Questionnaires and interviews among non-completers and non-participants of health surveys showed that reasons for not-completing the risk factor questionnaires included cognitive reasons such as (flawed) risk perceptions, health negligence, etc., while not participating in repeated tests was due to affective reasons such as “risk denial”, “fear of the outcome” and “fear of the consequences” (lifestyle changes and medication) [16, 17]. Still, there is a lack of knowledge about the extent to which demographic factors, lifestyle habits and physical factors are associated with non-participation in HRAs and repeated HRAs.

### Aim

The aim was to identify factors associated with not participating at all (in the initial HRA) as well as factors related to discontinuation of participation i.e. not participating in HRA follow-ups (repeated HRAs).

## Methods

### Setting

Data was collected in a longitudinal municipality-based health project conducted by an OHS in central Sweden, including one initial HRA, followed by actions on both individual and group level and two repeated HRAs, at 3 months and 9–10 months after the initial HRA, respectively, for evaluation of the effects. This study is a secondary analysis of the data that was primarily collected in order to study the effects of HRA.

#### The municipality-based health project

In a pilot study of 378 municipal employees who were followed over a 9-month period, it was found that, 53 out of the 233 employees (22%) from the “risk group” at initial HRA had moved to the “non-risk group” at the third repeated HRA. These positive results led to the implementation of this health project into all the remaining departments of the municipality between 2005 and 2007: Department for Childcare and Education, Department for Health and Social care, Department of Civil Administration, Department for Technical Service (including cleaning and construction), and Department for Municipal Council Management. The health project was funded by the municipality and carried out by a nation-wide privately-financed OHS. A project group was formed with representatives from both the OHS and the municipality. The leaders of the departments together with the administration managers and representatives from human resources were responsible for informing their employees about the health project and stimulating participation. The OHS was provided with a list of employees employed in each department and these were contacted individually by the OHS personal. The employees were offered a wide range of dates and times that they could choose from during working hours for participation in the initial and repeated HRAs. The HRA was free of charge for all employees. The employees were provided with a direct contact with the regular health care if a serious disease was detected. Seminars and health information on actions for healthier lifestyles were arranged and the employees were encouraged to participate in these seminars. Note, that this project was a clinical project and not registered as a clinical trial in any of the research data bases. The idea to perform research on this data came after the project was finished.

### Health risk assessment

The content of the HRA is described in previous reports [6–9]. HRAs follow a standardized procedure and are conducted in similar ways by the providers of OHS at the workplace. One HRA contains a self-administered questionnaire concerning physical and mental health (pain, medication and stress), lifestyle factors (physical activity, smoking, alcohol consumption and sleeping habits), as well as a number of physical measures, such as BMI, percentage of fat-free mass and cardiopulmonary fitness [18, 19].

The HPA includes three forms that participants fill in. The first is about health habits and perceived health, in which the participant answers a range of questions about health and lifestyle using one of five fixed options (1 = bad value and 5 = good value). The second is about physical measurements, while the third form is about setting personal goals. These measures are then used to classify an employee into either a “risk group” or “no-risk group”, based on predefined cut-offs on the variables from all forms. In a final step, employees are classified into an overall “risk group” if certain variables and combination of variables show an increased risk of future health problems and an individual action plan was set, including clear time-specified goals and methods for how to evaluate whether these goals were reached. With support from the physiotherapist, the participants from the “risk group” choose which actions they are going to work on over the next period of time, based on their individual goals. The actions could be walking, running and/or exercising at a gym (at an effort level corresponding to level 13 (“somewhat hard”) on Borg’s 6–20 RPE scale [20], change of diet, sleep habits, etc. Diaries, logs and calendars were often used to support these individual lifestyle changes [9]. All employees were invited to participate in the repeated HRAs three to six months later, i.e. also those from the non-risk group.

### Data analyses

#### Outcomes and exploratory variables

We used non-participation rates in the first and third HRA, respectively, as the binary outcome variables. The initial participation rate was calculated by dividing the number of employees with data from the initial HRA by the number of invited employees. For this outcome, only age, gender and department were available and used as explanatory variables.

The repeated participation rate was calculated by dividing the number of employees with data from three HRAs with the number of employees with data from only the initial HRA. For this outcome, all information available was used.

Data on the exploratory variables was collected in three different ways. Data on age, sex, department, and sickness absence was available on all employees and provided by the municipality.

- Age was categorized into four age categories (18–33, 34–45, 46–57, and 58–73 years).
- Sex was male or female.
- The department in which the employee was working in was used to categorize work type; employees from the Department for Childcare and Education were used as the reference category in the logistic regression analyses. Four other departments were used: the Department for Health and Social Care), the Department of Civil Administration, Department for Technical Service), and the Department for Council Management).
- Sickness absence was dichotomized based on a cut-off point of ≥14 days in the last year, i.e. the year before the date of participation, and data on this variable was available for only 1423 employees of the 2022 employees in the sample.

Lifestyle and health-related data was provided by the employee during their HRA and was documented in a central database of the HPA by the HRA personnel. Hence, this information was only available for those who participated in HRAs. The employees were asked to respond to several 2 or 5-level multiple choice questions and other types of question, about lifestyle factors, such as tobacco and alcohol consumption, physical activity, nutritional habits, and health-related factors, such as usage of pharmacological drugs, the occurrence of pain/discomfort in different areas (neck/back, stomach) and sleeping problems, as well as questions on feeling lonely, stressed or exhausted. Self-rated health was assessed using the Swedish version of the EQ5D questions [21]. Based on an index of all these aspects of health and lifestyle, each individual was subsequently classified into an “overall health” index in which the employees were classified as “Having good health/lifestyle” or “Having not such good health/lifestyle” as described in previous studies [9].

- Employees were asked whether they smoked tobacco or used snuff. They were classified as ‘smokers’ or ‘snuff users’ if they responded affirmatively about daily or irregular smoking/snuff use.
- Risky drinking was assessed based on three questions concerning alcohol consumption (AUDIT-C), a reliable short form of the Alcohol Use Disorders Identification Test (AUDIT), which includes frequency over the past 12 months, typical quantity and binge drinking, for a maximum score of 12 (responses ranging from 0 to 4, a higher score indicating a greater risk). The cut-offs for risky drinking were set at 6 for men and 4 for women in accordance with a validation study of AUDIT-C compared to full AUDIT in occupational health care [22].
- The employees were classified as being ‘physical inactive’ if they responded ‘less than 1 hour a week’ or ‘between 1 and 3 hours per week’ to the questions regarding the number of hours with moderate or vigorous activity per week, as defined by Swedish recommendations [23].
- The employees were classified as having an “inactive leisure time” if they answered “never”, “very rarely” or “rarely” to at least one of the questions regarding visiting art exhibitions, theatres, cinemas, sports events, restaurants, families and friends.
- Based on an index of 0.07–6 that has previously been used in Swedish public health research, two questions explored nutritional habits in terms of vegetable, fruit and berry consumption, assessed as ‘inadequate’ (index < 1.3) or ‘adequate’ (> 1.3) [24].
- The Shirom-Melamed Burnout Measure, consisting of 14 questions, was used to rate employees as ‘stressed out’ if they scored 4.0 or higher and ‘non-stressed out’ for ‘little’ or ‘not at all’ [25].

Finally, the OHS personnel completed this database with data from physical measurements collected in a clinical setting. These measures were dichotomized using the standard cut-offs for classifying being at risk or not and described elsewhere [9, 26]. An individual was classified as being “at risk” when they

- had a blood pressure (either systolic or diastolic pressure above 149 and 90, respectively), or were taking blood pressure medicines
- were diagnosed with diabetes
- were diagnosed with asthma
- were classified as overweight or “obese”, i.e. a body mass index (BMI) > 25 and > 30, respectively. BMI was calculated as weight (in kg) / height^2^ (in m)
- had poor cardiovascular fitness according to Åstrand [17, 18]
- had a V0_2_ capacity below average (according to Åstrand) [17, 18].

#### Logistic regressions

For each outcome variable, a univariate logistic regression was performed per protocol (excluding those with incomplete data) in order to assess the odds ratio (OR) for the association between the independent variable and the outcome (non-participation in an HRA). For those variables with significant (*p* <  0.05) univariate associations, a multiple logistic regression analysis was performed, resulting in adjusted OR’s for all variables included. IBM SPSS for Windows, version 24 was used for all calculations.

## Results

### Non-participation rate

Of the 2022 employees asked to participate in the project, 1104 (54.6%) did not participate in the initial HRA. Of the 918 people who participated in at least one HRA, 351 (38.2%) did not participate in three repeated HRAs.

### Factors associated with non-participation in initial HRA

Table 1 provides the results from the uni- and multiple analysis. In the univariate analysis, all four factors that were tested were significantly associated (*p* <  0.05) with non-participation in the initial HRA, and therefore were further included in a multiple regression model. All four variables remained significant in the final model. Non-participation was associated with younger age; 18–33 years (OR = 3.76; 95%CI = 2.53–5.59), and male sex (OR = 1.87; 95%CI = 1.36–2.58), as for employees working at the Department for Technical Service (construction workers/cleaners/ technicians) (OR = 9.00; 95%CI = 5.92–13.70) or employees working at the Department for Health and Social care (health care workers) compared with employees from the Department for Childcare and Education (OR = 2.62; 95 %CI = 2.32–3.72). Finally, sickness absence for 14 days or more during the course of the year was also a significant factor associated with non-participation, (OR = 1.50; 95%CI = 1.10–2.20).

**Table 1:** Odds ratios and 95% confidence intervals (95%CI) for associations with non-participation in an initial HRA

	Univariate Model^a)^				Multiple Model^b)^			
	n	Non-participators (%)	OR^c)^ (95% CI)	*p*-value	n	Non-participators (%)	OR^c)^ (95% CI)	*p*-value
Age								
58–73	536	46	1		399	36	1	
46–57	569	46	1.01 (0.80–1.28)	0.919	458	37	0.95 (0.70–1.29)	0.759
34–45	496	50	1.14 (0.90–1.16)	0.285	378	38	1.17 (0.85–1.60)	0.344
18–33	421	82	**5.27 (3.90–7.12)**	**< 0.001**	182	67	**3.76 (2.53–5.59)**	**< 0.001**
Sex (female)								
Female	1621	52	1		1147	39	1	
Male	401	63	**1.57 (1.25–1.96)**	**< 0.001**	127	51	**1.87 (1.36–2.58)**	**< 0.001**
Department								
Department for Childcare & Education	608	37	1		446	26	1	
Department for Health & Social care	861	63	**2.94 (2.40–3.69)**	**< 0.001**	582	47	**2.62 (1.97–3.48)**	**< 0.001**
Department of Civil Administration	213	43	1.30 (0.95–1.79)	0.102	150	21	0.82 (0.52–1.29)	0.384
Department for Technical Service (including cleaning & construction)	216	81	**7.10 (4.88–10.33)**	**< 0.001**	172	18	**9.00 (5.92–13.70)**	**< 0.001**
Department for Municipal Council Management	62	48	1.6 (0.94–2.73)	0.082	47	38	1.82 (0.96–3.45)	0.069
Sickness absence								
No	1180	39	1		1174	39	1	
Yes	243	49	**1.50 (1.14–1.98)**	**0.004**	243	49	**1.50 (1.10–2.00)**	**0.004**

^a^The univariate model shows the association between one variable and outcome at the time

^b^In the multiple model, all included variables are adjusted for each other

^c^OR expressed in bold indicates significant associations

### Factors associated with non-participation in repeated HRAs

For those who participated in the initial HRA, seven out of 28 variables were in the univariate analysis significantly (*p* <  0.05) associated with non-participation in the follow-up HRA (Table 2). These were included in a multiple regression model for further analyses. The results showed that four variables remained significant. Age was associated with non-participation in repeated HRAs; increased ORs were found for those between 18 and 36 years (OR = 2.05; 95%CI = 1.13–3.73) and for those between 37 and 47 years (OR = 1.53; 95%CI = 1.03–2.28) in comparison with those with higher ages. Associations with non-participation were also found for different departments; employees from the health and social care (OR = 1.93; 95%CI = 1.38–2.71), civil administration (OR = 2.56; 95%CI = 1.63–4.01), and employees at the technical departments were less likely to participate as those at the childcare and education department (OR = 4.47; 95%CI = 2.24–8.93). Finally, those who were on sick leave for 14 days or more during the course of the year (OR = 1.50; 95%CI = 1.00–2.24), were less likely to participate in repeated HRAs (OR = 1.50; 95% CI = 1.00–2.24). None of the physical measured variables significantly increased the odds of non-participation. Among the self-rated life style variables, only reporting inadequate nutritional habits (OR = 1.42; 95%CI = 1.02–1.99) was associated with non-participation and more likely not to participate in repeated HRAs.

**Table 2:** Odds ratios and 95% confidence intervals (95%CI) for associations with non-participation in repeated HRAs

			Univariate Model^a)^		Multiple Model^b)^	
	Non-participators		OR (95% CI)^c)^	*p*-value	OR (95% CI)^c)^	*p*-value
	n	(%)				
Age						
58–73	257	30	**1**		1	
48–58	278	39	**1.49 (1.04–2.14)**	**0.030**	1.26 (0.87–1.84)	0.222
37–47	259	41	**1.68 (1.16–2.41)**	**0.005**	**1.53 (1.03–2.28)**	**0.035**
18–36	124	49	**2.31 (1.48–3.59)**	**< 0.001**	**2.05 (1.13–3.73)**	**0.018**
Sex (female)						
Male	771	38	1			
Female	147	41	1.14 (0.79–1.63)	0.482		
Department						
Childcare & Education	384	28	**1**		**1**	
Health & Social care	315	44	**2.02 (1.47–2.76)**	**< 0.001**	**1.93 (1.38–2.71)**	**< 0.001**
Civil Administration	121	46	**2.20 (1.45–3.35)**	**< 0.001**	**2.56 (1.63–4.01)**	**< 0.001**
Technical Service (including cleaning & construction)	42	62	**4.15 (2.14–8.05)**	**< 0.001**	**4.47 (2.24–8.93)**	**< 0.001**
Municipal Council Management	31	26	0.89 (0.39–2.05)	0.782	0.97 (0.39–2.35)	0.922
Sick leave						
No	715	38	**1**		**1**	
Yes	123	48	**1.60 (1.13–2.45)**	**0.010**	**1.50 (1.00–2.24)**	**0.050**
Self-ratings						
Smoking						
Non-smokers	753	36	**1**		1	
Smokers	165	49	**1.67 (1.19–2.35)**	**0.003**	1.43 (0.97–2.10)	0.069
Alcohol						
Non-risky drinkers	880	38	1			
Risky drinkers	38	45	1.32 (0.69–2.55)	0.401		
Physical activity						
Active	626	36	**1**		**1**	
Non-active	292	44	1.41 (1.06–1.87)	0.017	1.33 (0.96–1.85)	0.086
Leisure time						
Active	836	38	1			
Non-active	82	39	1.04 (0.65–1.65)	0.878		
Nutrition						
Adequate	616	34	**1**		**1**	
Inadequate	302	46	**1.66 (1.25–2.20)**	**< 0.001**	**1.42 (1.02–1.99)**	**0.037**
Medicine						
Use of Analgesics						
No	831	39	1			
Yes	87	36	0.88 (0.56–1.40)	0.600		
Use of Sleeping pills						
No	903	38	1			
Yes	15	47	1.42 (0.51–3.96)	0.500		
Use of Gastrointestinal medicine						
No	780	42	1			
Yes	26	54	1.59 (0.73–3.49)	0.246		
Use of Mood regulators						
No	881	38	1			
Yes	37	51	1.75 (0.90–3.73)	0.098		
Discomfort/Pain						
No	750	39	1			
Yes	168	36	0.90 (0.639–1.28)	0.570		
Neck and back problems						
No	735	38	1			
Yes	183	40	1.09 (0.78–1.52)	0.607		
Sleeping problems						
No	830	38	1			
Yes	88	45	1.38 (0.75–1.55)	0.881		
Stomach problems						
No	843	38	1			
Yes	75	45	1.38 (0.86–2.21)	0.188		
Loneliness						
No	878	38	1			
Yes	40	35	0.864 (0.45–1.68)	0.667		
Stress						
No	662	38	1			
Yes	256	40	1.12 (0.84–1.51)	0.438		
Exhaustion						
No	777	38	1			
Yes	141	40	1.08 (0.75–1.55)	0.694		
Perceived health						
Good	630	34	1			
Bad	288	41	1.16 (0.87–1.54)	0.314		
Overall Health index						
Non-risk group	349	34	**1**		1	
Risk group	569	41	**1.33 (1.01–1.76)**	**0.044**	1.06 (0.75–1.50)	0.757
Physical measures						
High blood pressure						
No	832	39	1			
Yes	86	33	0.76 (0.47–1.22)	0.256		
Diabetes						
No	901	38	1			
Yes	17	35	0.88 (0.32–2.40)	0.801		
Asthma						
No	865	38	1			
Yes	53	36	0.90 (0.50–1.60)	0.713		
BMI						
< 25	424	37	1			
25–30	364	40	1.15 (0.86–1.53)	0.345		
> 30	128	39	1.11 (0.74–1.67)	0.607		
Cardiovascular test value						
Good fitness	597	36	1			
Poor fitness	149	43	1.35 (0.94–1.94)	0.109		
Physical performance VO_2_ max						
Good fitness	402	37	1			
Poor fitness	112	41	1.21 (0.80–1.82)	0.367		

^a^The univariate model shows the association between one variable and outcome at the time

^b^In the multivariate model are all included variables adjusted for each other

^c^Odds Ratios (OR) expressed in bold indicates significant associations

## Discussion

Of the employees who were asked to participate in the health project, more than half did not participate in any HRA and of those who did, more than one third did not participate in their follow-up HRAs. Younger age and male sex were factors associated with non-participation in an initial HRA, together with being on sick leave for 14 days or more during the last year. Moreover, employees from the technical department, the department of health and social care, and the department of municipal council management and were less likely to participate compared to the employees from the childcare and education department. These factors, together with having “poor dietary habits”, were also associated with discontinuation of participation, i.e. non-participation in repeated HRAs.

### Non-participation rates

The non-participation rate for participating in an initial HRA in this study was 54.6% and similar non-participation rates were found in previous studies [11, 12]. Interestingly, compared to these non-participation rates, the non-participation rate of repeated HRAs decreased (38.2%), but was still relative high. Out of all 2022 employees, only 567 (28.0%) participated in all three HRAs. This means that OHS not only need to work on convincing employees to participate in HRAs in general but needs to continue to work on keeping it relevant enough for employees to consider continuing using them.

### Non-participation in initial HRA

The finding that male sex and younger age were associated with non-participation were in accordance with previous studies [11, 13]. However, the highest participation of the employees from the Department for Childcare and Education compared to other departments was somewhat unexpected. Since the majority of these employees are teachers and other occupations with academic degrees, one could expect that these employees has a greater health perception compared to, e.g. the cleaners and construction workers from the technical department. Cognitive factors such as health perception have great influence on participation [16]. This finding could also be dependent on sex- and age differences between the departments, but, on the other hand, adjustments for sex and age were made in the regression models. One other plausible explanation for the difference between the departments could also be that the information about the health project might have been provided differently by the different heads of the departments. Employee participation in health promotion programs has been shown to be related to the degree of engagement by the employer [27] and other organizational factors [28]. Non-participation can be partly explained by moral issues, such as having reluctance against the employer interference with the employee health and that employees preferred to keep private life and work separate [15]. When employers are engaged in integrated workplace health promotion programs which focus on both lifestyle and work factors, both the employee and the employer are expected to take action to stay in good health and this could lower the resistance to employer interference [29]. Moreover, the contract between the employer and the health program provider should also contain information on how the results are going to be used, how the employees are followed over time, and how this information is reported to all partners.

The lower participation rate for employees with sickness absence > 14 during the year before the invitation could on one hand be explained by the fact that these employees were not reached by the invitation or on the other hand by the fact that research has shown that those with frequent contact with the regular health care system are less willing to participate in health screening projects [11].

The practical implication of the present study could be that employers should work systematically and could improve participation rates if special attention to the identified groups is given. There exists a vast amount of different health promotion strategies to promote a healthy lifestyle at the workplace. Besides introducing different incentives like co-payment [30], Pronk & Kottke [31] put forward three principles to increase physical activity in work places: 1) Create inter-relationships of individuals and their work environment; 2) Prioritizing evidence-based interventions, and 3) Aligning selected interventions with best practices for comprehensive worksite health promotion programs. This seems to be valid for the promotion of HRA as well. In practice, this means that employers that want to integrate HRA’s in their company, should identify the target groups (if not including all employees), choose the (evidence-based) HRA that is aligned to their company, and translate the results from the HRA’s (and repeated HRA’s) into actions at individual-, group- and company level. Rather looking to absolute differences on group level over time, we believe that studying relative differences on individual level could be more successful. The use of a specific questionnaire (the WELCOA Needs and Interest Survey) are suggested, which could be a useful tool to gauge employee interest in workplace wellness programs [32].

### Non-participation in repeated HRAs

To our knowledge, this is the first study that has tried to study factors associated with repeated HRAs. We hypothesized a selected compliance with repeated HRAs. We expected that, for instance, those with “poor health/lifestyle” at baseline were unlikely to participate in a follow-up HRA, especially if they knew that they had not succeeded in making any health/lifestyle changes, compared to those who had succeeded in making lifestyle/health changes. On the other hand, those with “poor health/lifestyle” at baseline should have received more attention from the HRA personnel during the two sessions. Previous studies have indicated that only the healthiest employees tend to participate in health programmes [28, 33], but our findings showed that factors other than good health/good lifestyle habits per se were more important for non-participation in repeated HRAs, i.e. age and the department. Our results that some departments are more likely to participate than other departments, might imply it is important that health projects should have a structured communication plan with continuous process evaluation and follow-ups, so all employees receive similar information.

### Limitations and strengths of the study

The primary goal of the data collection in this municipality health project was to study the effect of HRAs and repeated HRAs, but, due to the large number of drop-outs (only 28% from the original population provided data of baseline and follow-up HRAs) and the lack of a control group, it was not possible to draw any conclusions on the effectiveness of this health project. Instead, we used the data to study the factors associated with non-participation in this secondary analysis.

One strength of this study is that our study mirrors a “real” health promotion invention that was performed without interference of any researcher. We believe that if this project was performed as a prospective clinical trial, the participation rates could have been biased.

One of the limitations of this study was that information of sickness absence was not available for all invited employees and resulted in the loss of individuals in the multiple regression analyses. However, this lack of information was similar across age groups, sex, and departments and should not have influenced the estimates to a great extent, rather only the narrowness of the 95%CIs. Moreover, the use of self-ratings for assessing life-style and health-related variables has earlier been criticized due to the risk of overestimation of healthy life styles [34], but this should not have affected the results on participation.. Additionally, the invitation to participate in HRAs and repeated HRAs was provided by the head of the department and could have differed between the different departments. However, including employees through heads of the departments is the method used by the OHS and mirrors the “real” world, rather than the situation in a research design, and could be seen as a strength of the study, together with the large sample size, the longitudinal design, and the robust statistical models that were used. Finally, the present study was conducted in Sweden, which in one way could hamper the generalizability to countries in which occupational health services are organized differently or to countries with different social norms or economic pressure. On the other hand, the wide range of employees included in the study can be seen as a strength of this study.

### Further studies

Further studies with different approaches (randomized controlled studies, and qualitative designed interview/focus group-studies) are needed in order to understand why employees choose or choose not to participate in HRAs. This knowledge could then give suggestions for adapting the HRAs so as to increase the participation rates in the future.

## Conclusions

This study confirmed the low participation rates in HRAs found in previous studies. It also confirmed the age and sex-related non-participation factors of previous studies. Concerning repeated HRAs, the present study indicates that the factors age, sex, department, sick leave in the previous year and self-rated inadequate nutrition lifestyle are associated with discontinuation of participation i.e. non-participation in repeated HRAs. The results of this study could be of value for further discussions between OHS providers and employers with the aim of increasing participation focusing specifically on optimizing conditions and adjusting communication/motivation strategies for groups with high rates of non-participation. Future studies should also focus on modifiable variables that could make the HRAs more attractive and inclusive.

## Abbreviations

95% CI: 95% Confidence Interval
AUDIT: Alcohol Use Disorders Identification Test
BMI: Body Mass Index
HPA: Health Profile Assessment
HRA: Health Risk Appraisal
OHS: Occupational health services
OR: Odds Ratio
